# Prognosis Prediction Using Therapeutic Agreement of Video Conference–Delivered Cognitive Behavioral Therapy: Retrospective Secondary Analysis of a Single-Arm Pilot Trial

**DOI:** 10.2196/15747

**Published:** 2019-11-15

**Authors:** Kazuki Matsumoto, Tokiko Yoshida, Sayo Hamatani, Chihiro Sutoh, Yoshiyuki Hirano, Eiji Shimizu

**Affiliations:** 1 Research Center for Child Mental Development Chiba University Chiba Japan; 2 Japan Society for the Promotion of Science Chiba Japan; 3 Department of Cognitive Behavioral Physiology Graduate School of Medicine Chiba University Chiba Japan

**Keywords:** therapeutic alliance, cognitive behavioral therapy, obsessive-compulsive disorder, panic disorder, social anxiety disorder, video conferencing

## Abstract

**Background:**

The therapist-patient therapeutic alliance is known to be an important factor in cognitive behavioral therapy (CBT). However, findings by previous studies for obsessive-compulsive disorder (OCD), panic disorder (PD), and social anxiety disorder (SAD) have not been consistent regarding whether this alliance provides symptomatic improvements.

**Objective:**

This study investigated predictors of symptom improvement in patients receiving CBT via video conferencing.

**Methods:**

A total of 29 patients who participated in a previous clinical trial were recruited for the current study. Therapeutic alliance and clinical background in patients with OCD, PD, and SAD were measured at first session or the eighth session, which were calculated by multiple regression analyses to estimate the impact on therapeutic response percentage change.

**Results:**

The multiple regression analyses showed that, among the independent variables, only patients’ agreement in the therapeutic alliance remained viable, as other variables were a best fit for the excluded model (*P*=.002). The results show that patients’ agreement on therapeutic goals and tasks explains the prognosis, as the normalization factor beta was 0.54 (SE 32.73; 95% CI 1.23-5.17; *P*=.002) and the adjusted *R*^2^ was .266.

**Conclusions:**

Patients' agreement on therapeutic goals and tasks predicts improvement after CBT via video conferencing.

**Trial Registration:**

UMIN Clinical Trial Repository UMIN000026609; https://tinyurl.com/ye6dcbwt

## Introduction

One of the principles of cognitive behavioral therapy (CBT) is the therapist-patient therapeutic relationship [1], which is consistently important from the initial session of therapy to its last stage [2-4]. The therapist and patient collaboratively work together over time on the patient’s therapeutic goal of achieving symptomatic relief. However, the results of previous studies have not been consistent about whether the therapeutic relationship in CBT affects symptomatic outcomes [4,5].

Two systematic reviews, including a meta-analysis of depression, have shown moderate correlations between the therapeutic relationship (assessed through the Working Alliance Inventory [WAI] scale) and symptomatic outcomes [6,7]. These previous studies, conducted by correlation analysis, could not explain the causal relationship. Further, the causal relationship between the therapeutic relationship and its outcomes was discussed by retrospective observational studies in clinical trials, but the results were not consistent. For example, in CBT used for the treatment of panic disorder (PD), it has been suggested that a high rate on the WAI-Short Form (WAI-SF) is an important factor for a patient’s symptomatic improvements [8]; however, regarding CBT for the treatment of obsessive-compulsive disorder (OCD), it has been reported that scores on the WAI-SF did not affect the patient’s symptomatic improvements [9]. Furthermore, in a recent study of the WAI-SF [10], the two factors of therapeutic relationship and patient agreement were analyzed. It was found that a patient's strong agreement with CBT tasks predicted symptomatic improvements. In the past, the WAI-SF was supposed to have three factors: development of an affective bond, agreement with the task, and agreement with the goal between the therapist and patient [10]. CBT requires a restructuring of dysfunctional cognitions and behaviors that a patient has formed over many years; thus, it seems logical to infer that patients' agreement may have an impact on symptomatic improvements.

Use of the internet has spread worldwide and it has seen use by 4.536 million people (58.8%) globally as of June 2019 [11,12]. Internet-based CBT was created as a result of the incorporation of programming technology into CBT, and it has demonstrated effective results through its therapeutic processes by creating symptomatic improvements [13,14]. While the telemedicine approach is the most like traditional face-to-face treatment, previous research on video conference–delivered CBT has been limited compared to normal internet-based CBT [15-20]. A systematic review revealed that the therapeutic relationship is maintained at sufficiently high levels when using video conferencing [21]. However, to the best of our knowledge, the influence of the therapist-patient relationship developed through video conference–delivered CBT on patients’ symptomatic improvement has not yet been investigated [22]. In the current study, we investigated predictors for improvement among patients after they received CBT via video conferencing.

## Methods

### Study Design and Participants

This study utilized secondary data analysis with data from a previous clinical, pilot, single-arm trial on video conference–delivered CBT, using Cisco WebEX as the video conferencing system [19]. A total of 29 Japanese adult participants (mean age 35.5 years old; SD 9.2), 5 of whom were male and 24 of whom were female, completed the intervention. We hypothesized that therapist-patient agreement on therapeutic goals and challenges would predict patient prognosis. Baseline data was used exploratorily and analyzed through a series of statistical analyses.

### Measures

To assess the severity of symptoms as the primary outcome, we evaluated each mental health disorder of interest using a corresponding scale: OCD was assessed through the Yale-Brown Obsessive-Compulsive Scale (Y-BOCS); PD was assessed through the Panic Disorder Severity Scale (PDSS); and social anxiety disorder (SAD) was assessed through the Liebowitz Social Anxiety Scale (LSAS) [23-25]. Furthermore, depression was assessed through the Patient Health Questionnaire-9 (PHQ-9) [26], general anxiety was assessed through the Generalized Anxiety Disorder-7 (GAD-7) scale [27], and the therapist-patient therapeutic relationship was assessed through the WAI-SF [2]. Regarding the WAI-SF sub-scales, the agreement score was composed of the total scores of items 1, 2, 6, 8, 11, and 12, and the bond score was composed of the total scores of items 3, 5, 7, and 9 [10].

In our previous clinical trial, CBT was evaluated at the first, eighth, and sixteenth session [20], but the therapeutic alliance per WAI-SF [2] was set as a predictor in the eighth session. This is because it is anticipated that a well-established treatment relationship in the first half will affect the patient's engagement with the second half of the challenge (mostly with exposure). Depressive symptoms in PHQ-9 and general anxiety in GAD-7 at baseline (first session) were also set as predictors.

### Statistical Analysis

The statistical analysis was performed using SPSS Statistics, version 24.00 (IBM, Armonk, New York, United States). First, Spearman correlation analysis was performed between the treatment response percentage change and the score in each scale (WAI-SF total, PHQ-9, GAD-7), or the subscales of WAI-SF (agreement, bond). Second, to investigate the predictive effects that the patients' backgrounds at pretreatment may have had on the treatment response change post treatment, a series of multiple regression analyses were performed. The treatment response percentage change was set as a dependent variable in multiple regression analyses. Variables were entered for analysis in a multivariate model by the forward selection stepwise procedure (*F*<0.05 as inclusion and *F*≥0.10 as exclusion). Multicollinearity was measured by variance inflation factors (VIF) and tolerance. If the VIF value exceeded 4.0, or by tolerance was less than 0.2, then there was a problem with multicollinearity [28]. We excluded WAI-SF’s total scores because VIF was 13.09. The treatment response percentage change was calculated by dividing the total pretreatment score with the score difference between Session 1 and Session 16. The treatment response percentage change in this study was the decline in baseline Y-BOCS, PDSS, or LSAS score. The degree of treatment response percentage change was analyzed as a continuous variable and calculated as follows:


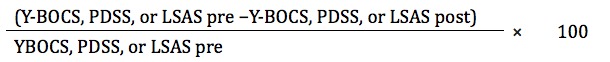


## Results

Agreement, bond, and the total score of the WAI-SF showed a significant correlation with the response percentage change as a result of correlation analysis, including Bonferroni correction (Table 1). Multiple regression analyses showed that, within the independent variables only patient agreement remained, with the other variables a better fit for the excluded model (*beta*=.54; Adjusted *R^2^*=.266; SE 32.73; 95% CI 1.23-5.17; *P*=.002).

**Table 1:** Correlation relationship between response percentage change and the patient’s background/symptoms.

Variable	Response percentage change (*r*)	*P* value	*P* value after Bonferroni correction
Patient agreement	.681	<.001	.005
Bond	.476	.009	.05
WAI-SF^a^ total	.569	.001	.005
PHQ-9^b^	–.228	.23	>.99
GAD-7^c^	–.292	.12	.60

^a^WAI-SF: Working Alliance Inventory–Short Form.

^b^PHQ-9: Patient Health Questionnaire-9.

^c^GAD-7: Generalized Anxiety Disorder-7.

Using G*power 3.1 [29] for power analysis, power (1–beta error probability) was calculated to be 0.88 (effect size *f*^2^=.36; alpha error probability=.05; sample size=29), and the number of predictors was one.

## Discussion

### Primary Findings

The hypothesis of this study was that agreement on therapeutic goals and challenges predicts a patient’s prognosis. We performed multiple regression analyses on the variables that were significantly correlated, but only the explanatory model using the patient agreement variable from the WAI was the best fit. The results suggested that patients’ agreement with the set goals and tasks during the middle stage of CBT predicted symptomatic improvement.

These results are consistent with a previous study, which provides evidence that the therapist-patient therapeutic relationship is important to symptomatic improvements in the middle to late stages of therapy [4,7]. Conversely, the results of a meta-analysis of guided, internet-based CBT (except by video conferencing) suggested that the therapeutic alliance is not important to the improvement of anxiety [5]. It is interesting that the results of the current research contrast with these results from a previous study, indicating that perhaps patients who do not agree with treatment drop out early. Thus, the therapist-patient therapeutic treatment alliance in internet-based CBT may not be relevant to the therapeutic response. The total WAI score was associated with symptomatic improvements when the treatment was tailored specifically to the patient's condition during internet-based CBT [6]. Results from this prior research provided important knowledge on future directions CBT could take using the internet. Specifically, it is essential to adhere to the content of basic CBT skill sets [30]. Hence, we can infer that the therapist’s work in implementing a personalized therapy may result in the patient's agreement with the therapeutic goals and tasks. Furthermore, this study’s results did not identify a pretreatment predictor, consistent with previous studies of depression [31].

### Limitations

First, this study had a small sample size, so future studies with a more significant sample size are needed. Second, factors affecting treatment responsiveness may be influenced by the quality of CBT, which can be assessed using a cognitive therapy scale, as well as a patient’s background and their relationship with the therapist [32,33]. This study does not assess quality of treatment, patient background, or relationship with the therapist, therefore, further studies exploring this aspect of the treatment are needed. Finally, this study was a secondary analysis of a single-arm pilot study.

### Conclusions

Our results suggest that patients' agreement with therapeutic tasks and goals predicts an improvement after intervention with video conference–delivered CBT. To the best of our knowledge, this is the first published evidence of this phenomenon. Therapists in the video conference–delivered CBT field should seek ways to apply tasks and goals that are tailored specifically to each patient's condition.

## Abbreviations

CBT: cognitive behavioral therapy
GAD-7: Generalized Anxiety Disorder-7 scale
LSAS: Liebowitz Social Anxiety Scale
OCD: obsessive-compulsive disorder
PD: panic disorder
PDSS: Panic Disorder Severity Scale
PHQ-9: Patient Health Questionnaire-9
SAD: social anxiety disorder
VIF: variance inflation factor
WAI: Working Alliance Inventory
WAI-SF: Working Alliance Inventory–Short Form
Y-BOCS: Yale-Brown Obsessive-Compulsive Scale
